# The comparison of perceived health-related quality of life between Australian children with severe specific language impairment to age and gender-matched peers

**DOI:** 10.1186/s12887-018-1058-2

**Published:** 2018-02-14

**Authors:** Kristy Nicola, Pauline Watter

**Affiliations:** The Univeristy of Queensland, Brisbane, QLD 4072 Australia

**Keywords:** Health-related quality of life, The pediatric quality of life inventory™ version 4.0 generic core scales, Specific language impairment, Children, Quality of life

## Abstract

**Background:**

Children with specific language impairment often present with multiple comorbidities, which may adversely affect both participation in play and academic performance, potentially impacting a child’s health-related quality of life. This study 1) explored the suitability of the Pediatric Quality of Life Inventory™ Version 4.0 Generic Core Scales (PedsQL™) for use with a typically developing Australian control group, and 2) compared the health-related quality of life between a control group and Australian children with severe specific language impairment.

**Methods:**

Health-related quality of life data collected as part of a broader study of 43 children with severe specific language impairment (males = 35, age range 5–16, mean age = 8.79+/− 2.92) enrolled at a special school were used to explore previously unreported findings. Typically developing gender and age matched (+/− 3 months) peers were recruited from local schools. The PedsQL™ child self-report and proxy-report were individually or interviewer-administered to the control group as required, and then compared to the group with specific language impairment.

**Results:**

The PedsQL™ was reliable and feasible for use with the control group (*N* = 43, males = 35, age range = 5–16 years, mean age = 8.74+/− 2.94 years). Control group performance was as expected as per the manual. Parents of the control group scored their children significantly higher than did the children themselves on all scales except the emotional functioning scale. Both the control group children and their parents scored themselves significantly higher on all scales, compared to children with severe specific language impairment and their parents.

**Conclusions:**

The PedsQL™ was suitable for use with the control group. Further, the recruitment of a control group provided additional clarity on the extent a severe specific language impairment impacts on an Australian child’s perceived health-related quality of life, compared to the manual cut-off scores. Severe specific language impairment significantly impacts negatively on the health-related quality of life of Australian children across all domains, particularly when compared to an age and gender-matched group of peers. These results warrant the inclusion of health-related quality of life evaluations in the assessment of these children along with a multidisciplinary approach.

## Background

Within the literature, exploring the health-related quality of life (HRQOL) from the perspectives of children with specific language impairment (SLI), and their parents is in its infancy. HRQOL, is the term that embraces an individual’s perspective of how illness/injury, medical treatment and/or health care policy impact his/her own life (including physical and psychosocial dimensions) [1]. SLI affects approximately 7% of school-aged children [2, 3] and requires children to achieve an average score on nonverbal intelligence, while performing below average on standardized tests of receptive and/or expressive language ability [2, 3]. A mild presentation of SLI was reported to impact on the sleep and speech domains of the 17D questionnaire, but failed to impact the overall HRQOL score [4]. However, a severe SLI presentation was reported to impact the overall HRQOL score of the Pediatric Quality of Life Inventory™ Version 4.0 Generic Core Scales (PedsQL™) and particularly the social and physical functioning scales [5]. Such emerging findings warrant further exploration.

A rather evident issue in exploring the HRQOL of children with SLI evolves around the capacity of these children to reflect and report their perspectives of their own HRQOL. Consequently, researchers and clinicians are left with the challenge of ascertaining when a proxy report may be more appropriate or reliable. When a self-report is chosen, a HRQOL measure that is suitable for use with children with SLI is required to foster gathering useful information. Yet, it has been reported that a HRQOL measure suitable for use within a paediatric population with speech and/or language impairments has not been established broadly [6], and specific measures exploring a specific condition such as SLI similarly fail to exist. A lack of clarity in suitable measures may explain the absence of publications.

The authors of this paper reported the HRQOL of a group of Australian children enrolled in a school dedicated to the educational and therapeutic needs of children with severe speech and/or language impairments [5]. After exploring the literature and available HRQOL measures, the PedsQL™ [7] was selected and then confirmed to be suitable for use with the study participants [5]. As a starting point, it was valuable to confirm the suitability of the PedsQL™ in order to identify another potential measure appropriate for use with this group of children. Nicola & Watter (2015) then compared the PedsQL™ results from both child self-report, and parent proxy-report to the cut-off scores published by Varni et al. (2003) in order to identify if children with severe SLI were at risk for impaired HRQOL. The cut-off scores provided by Varni et al. (2003) reflect the perspectives of a sample of children and their parents residing in The United States of America, and are the scores that fall 1SD below the mean on all scales. However, in order to clearly understand the impact a severe SLI has on children residing in Australia, it is paramount to now compare the outcomes to age and gender-matched Australian peers. Comparison to a healthy sample of Australian children will provide a clearer picture of any identified deficits and the extent of these deficits by minimizing both cultural and environmental influences. In addition, it has not yet been reported if the PedsQL™ is suitable for use with a healthy sample of Australian children. This study aims to fill these gaps by 1) examining the initial feasibility and reliability of the PedsQL™ for use in an age and gender-matched healthy control group comprising 43 school-aged typically developing Australian children, and 2) exploring the perceptions’ of the control group children and their parents to those of children with severe SLI and their parents. Since the PedsQL™ has been identified as a reliable and valid tool for administration with healthy children elsewhere [8], we expect the PedsQL™ will be appropriate for use with the control group in this study. Further, consistent with the literature, we anticipate children with severe SLI will report significantly lower HRQOL when compared to their peers.

## Methods

The University of Queensland’s Medical Research Ethics Committee granted ethical approval for administration of the PedsQL™ to both children with severe SLI and their parent, along with an age and gender-matched peer and their parent. The current study includes both the participants from a previous study (*N* = 43) [5], along with newly recruited age and gender-matched peers.

### Participants

#### Children with severe specific language impairment

The following summary describes the children with severe SLI included in the previous study [5]. Children aged 4 years and older enrolled at a special school were invited to participate if they met the following two inclusion criteria: 1) verified within the speech-langauge impairment category of the Education Adjustment Program developed by the Queensland Government in Australia [9, 10], and 2) confirmation that the failure of normal language development occurs in the absence of other possible causes (e.g. hearing impairment) consistent with a SLI. Children were excluded if they were verified within any other category such as Intellectual Impairment or Autistic Spectrum Disorder, or where the langauge impairment could be explained by a clear cause such as hearing impairment. The final study group included 43 children (males = 35) from 5 to 16 years (mean = 8.79+/− 2.92 years) and their parent [5].

#### Control group (age and gender-matched peers)

Children with no identified difficulties were recruited from local schools via posters and information packages sent home with age appropriate children. Control children were invited to participate if they matched the gender and age (+/− 3 months) of a child in the severe SLI group. Parents signed consent, while child assent was obtained. Children and their parents could either complete the questionnaires at The University of Queensland with a researcher present, or alternatively complete their questionnaires at home and return them by post. If completed at home, parents were provided with step-by-step instructions, which encouraged independent completion of questionnaires with parents completing their questionnaire before the child did. A researcher could be contacted for support if required. The questionnaires were interview-administered by research assistants/parents for all children aged 5–7 years. Children aged 8 years and over were encouraged to independently complete their own questionnaires. When children independently completed the questionnaires, they were given as much time as necessary and a researcher/parent was available to assist if required.

### Instruments

#### The pediatric quality of life inventory™ version 4.0 generic core scales (PedsQL™)

The PedsQL™ is one of the most commonly used measures for exploring the HRQOL in children [11]. Both the child self-report (for children 5–18 years old) [12] and the proxy-report (for children 2–16 years old) [13] have well-established validity and reliability. The PedsQL™ is a 23-item measure that includes multiple age versions of identical child self-report and proxy-report, differing only in developmentally appropriate language and/or tense [7, 14]. We used both the child self- and proxy-report for the 5–7 years (young child version), 8–12 years (child version), and 13–18 years (adolescent version). The PedsQL™ encompasses physical, emotional, social, and school functioning scales on which individuals must rate how much of a problem each item within a scale has been in the last month [7, 14]. A 5-point Likert scale is used for children aged 8 and over, while the measure is simplified to a 3-point Likert scale that is anchored to a pictorial representation to assist children 7 and under [7, 14]. Items within a scale are reverse-scored and linearly transformed to a 0–100 scale [7, 14]. The transformed item scores are averaged for each scale or a combination of scales, to produce the following summary scores: Psychosocial Health Summary Score (the mean of emotional, social and school functioning scales), and Total Summary Score (the mean of all scales) [7, 14]. Higher scores indicate better HRQOL [7, 14].

### Statistical analyses

Data were managed using SPSS version 22 [15]. To determine suitability of the PedsQL™ for a cohort of children, previous authors have consistently performed a series of statistical analyses [5, 8, 12, 13, 16]. First, initial feasibility and reliability for use of the PedsQL™ is established for a particular cohort of children, and then performance on the PedsQL™ is interpreted. The feasibility of the PedsQL™ was explored separately for the control group children and parents by calculating the percentage of missing values for each item [5, 8, 12, 13, 16, 17]. To explore the scale internal consistency reliability for both the control group self-report and proxy-report, a Cronbach’s alpha coefficient was calculated. Consistent with previous studies, this study required each scale’s reliability to be above 0.70 for group comparison, or 0.90 for analyzing individual patient scale scores [5, 8, 12, 13, 16]. The range of measurement for the control group was explored by investigating the distribution of scores by calculating the percentage of extreme range scores. Extreme range scores are the maximum possible score (ceiling effect = 100) and the minimum possible score (floor effect = 0), with ceiling/floor effects within 1–15% considered acceptable [5, 12, 13, 16, 18]. Descriptive statistics were explored for both the control group child and parent perceptions. Cut-off scores (1SD below the mean on all scales and summary scores) published by Varni et al. (2003) were used to identify if any of the control group’s scales or summary scores fall within the “at-risk” for impaired HRQOL category. The Mann Whitney U test for nonparametric analysis was used to compare the mean scores between: control children and their parents, control children and children with severe SLI, and finally, control parents with parents of children with severe SLI.

## Results

### Describing the control group

Similar, to the group of children with severe SLI [5] the control group consisted of 43 children (males = 35, 81%) from 5 to 16 years old (mean = 8.74+/− 2.94 years) and their parents. Only *n* = 4 control children completed their questionnaires at home without researcher supervision.

### Feasibility (missing item responses), internal consistency reliability, and range of measurement for the control group

The percent of missing items for the control group was 0.0% for both child self-report and parent proxy-report. The minimum reliability standard of 0.70 required for group comparisons was achieved for all child self-report and parent proxy-report scales (Table 1). All floor effects fell below the recommended 15%; however, only half of the ceiling effects met this standard (Table 1).

**Table 1:** Reliability and Percentage of Floor and Ceiling Effects for Control Group Child Self-Report and Parent Proxy-Report on PedsQL™^*^

Scale	Cronbach’s Internal Consistency Reliability Coefficient Alpha	Floor Effect^a^	Ceiling Effect^b^
Child Self-Report			
Total Summary Score	0.80		
Psychosocial Health Summary Score	0.79		
Physical Functioning	0.86	0	37
Emotional Functioning	0.85	0	9
Social Functioning	0.83	0	56
School Functioning	0.89	0	30
Parent Proxy-Report			
Total Summary Score	0.78		
Psychosocial Health Summary Score	0.76		
Physical Functioning	0.88	0	12
Emotional Functioning	0.82	0	5
Social Functioning	0.81	0	35
School Functioning	0.85	0	14

^*^The Pediatric Quality of Life Inventory™ Version 4.0 Generic Core Scales

^a^The percentage of participants that achieved the minimum possible score of 0

^b^The percentage of participants that achieved the maximum possible score of 100

### Child self-report and parent proxy-report results for the control group

Table 2 presents results of the child self-report and parent proxy-report. While group mean scores were in the expected normal range, compared to the mean cut-off scores published by Varni et al. (2003), a small percentage of individual children and parents reported functioning below the mean cut-off scores on most scales.

**Table 2:** PedsQL™^*^ Results for both the Control Group Child Self-Report and Parent Proxy-Report

Scale	Range	Mean (SD)	Cut-off scores^a^	N(%)^b^
Child Self-Report				
Total Summary Score	67.23–98.91	85.06 (8.27)	69.71	4 (9.30)
Psychosocial Health Summary Score	63.33–100	82.36 (9.83)	66.03	3 (7)
Physical Functioning	68.75–100	90.12 (8.65)	72.98	3 (7)
Emotional Functioning	45–100	77.21 (14.32)	59.57	2 (4.65)
Social Functioning	60–100	86.98 (13.23)	66.61	5 (11.63)
School Functioning	55–100	82.91 (12.35)	62.99	4 (9.30)
Parent Proxy-Report				
Total Summary Score	72.83–100	89.18 (7.07)	65.42	0 (0)
Psychosocial Health Summary Score	65–100	86.59 (9.22)	64.38	1 (6.67)
Physical Functioning	65.62–100	94.04 (8.03)	63.28	0 (0)
Emotional Functioning	50–100	79.30 (12.56)	63.29	4 (9.30)
Social Functioning	65–100	92.79 (10.71)	62.07	1 (6.67)
School Functioning	50–100	87.67 (12.55)	56.75	1 (6.67)

^*^The Pediatric Quality of Life Inventory™ Version 4.0 Generic Core Scales

^a^Mean cut-off scores published in [8]

^b^Number below the cut-off score (percentage)

### Comparing control child self-report to control parent proxy-report

There were significant differences between child self-report and parent proxy-report on the following scales: Total Summary Score (z = − 2.34, *p* = 0.02), Psychosocial Health Summary Score (z = − 2.00, *p* = 0.04), Physical Functioning (z = − 2.79, *p* < 0.001), Social Functioning (z = − 2.24, p = 0.02), and School Functioning (z = − 2.00, *p* = 0.04), with parents consistently scoring their children higher (better) on each scale (refer to mean values in Table 2). There was no significant difference on the Emotional Functioning scale.

### Comparing child self-report of the control group to the child self-report of children with severe specific langauge impairment

To facilitate comparison, the mean scores on each scale for children with severe SLI [5] are reproduced for the reader in Table 3, along with the mean scores of the control group. Across all scales, children in the control group scored themselves significantly better than children with severe SLI as follows: Total Summary Score (z = − 5.62, *p* < 0.001), Psychosocial Health Summary Score (z = − 5.00, *p* < 0.001), Physical Functioning (z = − 5.24, *p* < 0.001), Emotional Functioning (z = − 2.00, *p* = 0.04), Social Functioning (z = − 4.36, *p* < 0.001), and School Functioning scales (z = − 5.00, *p* < 0.001).

**Table 3:** PedsQL™^*^ Child Self-Report and Parent Proxy-Report for Children with Severe SLI and the Control Group

Scale	Range	Mean (SD) of Control Group	Mean (SD) of Group of Children with severe SLI^a^
Child Self-Report			
Total Summary Score	67.23–98.91	85.06 (8.27)	66.51 (15.36)
Psychosocial Health Summary Score	63.33–100	82.36 (9.83)	67.00 (22.19)
Physical Functioning	68.75–100	90.12 (8.65)	67.00 (22.19)
Emotional Functioning	45–100	77.21 (14.32)	68.26 (20.23)
Social Functioning	60–100	86.98 (13.23)	66.28 (23.83)
School Functioning	55–100	82.91 (12.35)	64.19 (16.40)
Parent Proxy-Report			
Total Summary Score	72.83–100	89.18 (7.07)	63.73 (15.37)
Psychosocial Health Summary Score	65–100	86.59 (9.22)	60.54 (14.84)
Physical Functioning	65.62–100	94.04 (8.03)	69.69 (20.21)
Emotional Functioning	50–100	79.30 (12.56)	63.14 (17.99)
Social Functioning	65–100	92.79 (10.71)	56.51 (19.13)
School Functioning	50–100	87.67 (12.55)	61.98 (16.26)

^*^The Pediatric Quality of Life Inventory™ Version 4.0 Generic Core Scales

^a^Mean scores published in [5]

### Comparing parent proxy-report of the control group to the parent proxy-report of children with severe specific langauge impairment

As before, to facilitate comparison a reproduction of parent proxy-report mean scores for parents of children with severe SLI [5] are presented along with the control mean scores, which both can be found in Table 3. Control parents scored their children significantly higher than parents of children with severe SLI across all scales as follows: Total Summary Score (z = − 6.84, *p* < 0.001), Psychosocial Health Summary Score (z = − 6.75, *p* < 0.001), Physical Functioning (z = − 5.88, *p* < 0.001), Emotional Functioning (z = − 4.23, p < 0.001), Social Functioning (z = − 7.06, *p* < 0.001), and School Functioning (z = − 6.13, *p* < 0.001) scales.

## Discussion

This study aimed to explore the initial feasibility and reliability of the PedsQL™ for use in a group of typically developing Australian children. The results demonstrated that the PedsQL™ child and proxy reports were feasible and reliable for use in a group of typically developing Australian children who were age and gender-matched to the experimental severe SLI group. As might be expected, the control group provided a range of responses with a small percentage of children and parents perceiving their child’s functioning to be below the mean cut-off scores [8], however; these scores failed to demonstrate any floor effect. In some scales, the ceiling effect percentages for the control group exceeded the 15% threshold, however, the navigation towards higher scores was considered acceptable as it was within the anticipated direction for the healthy control group. As was expected, consistent with previous findings [8], the control group children and their parents mean score for each scale did not fall below the identified “at-risk” cut-off scores previously published [8]. This reflects a healthy population and further supports the use of the PedsQL™ with typically developing Australian children. Consequently, this study supports utility of the PedsQL™ child self-report and proxy-report with school-aged typically developing Australian children.

A closer look at the control group results revealed that the parents consistently scored their children significantly better than their children did in all scales, with the exception of the emotional functioning scale. This outcome aligns with previous studies, as it is generally accepted within the literature that parents of healthy children typically score their children better than the children themselves [19, 20]. Further, there is emerging evidence that rating emotional functioning often produces the largest disagreement amongst a child and their parent, when compared to objective domains such as physical functioning [19–21]. Inconsistent with the literature, the emotional functioning scale demonstrated the most agreement amongst control children and their parents in this study. Recently, research has attempted to identify variables that contribute to parent/child discrepancies [19]. Younger children unable to express emotions, older children engaging in more activities outside the home environment and parents required to spend increased time with younger children are aspects that appear to influence agreement either negatively or positively. In this study, agreement with the emotional functioning scale, could be attributed to the mean age of our group. The mean age was 8 years, which is young enough to still require parental supervision, guidance and involvement; yet old enough to express emotional feelings verbally.

This study additionally aimed to compare the perceived HRQOL reported by both children with severe SLI and their parents to the control group children and their parents. The perceived HRQOL was significantly lower on all scales and summary scores compared with the control group children and their parents. These results further support the use of the PedsQL™ within the Australian culture, particularly as it clearly differentiates the HRQOL between typically developing children and those with severe SLI. Interestingly, parents of children with severe SLI had less parent/child discrepancies than our control group. In fact, our control group was significantly different for parent and child responses on all but one scale, whereas our experimental group was only significantly different on one scale – social functioning [5]. This may be reflected in both the characteristics of a severe SLI and the child him/herself. When an illness is active (vs inactive), it demands greater parent/child communication to address symptoms, resulting in higher concordance between parents and their children [19]. It is likely that a severe SLI demands greater parent/child communication, but for different reasons. For example, children with a severe SLI may have an increased dependency on their parents to both care for them and become their “voices”. Parents of a child with a severe SLI will often communicate on behalf of their child, or “translate” for their child in order to support, facilitate and at times allow for communication with others to occur. Consequently, parents of children with severe SLI may spend more time with their child, regardless of age, and be more involved in their child’s daily interactions in order to provide support. In addition to this close relationship, it is likely that children with severe SLI are not engaging in a variety of activities outside of the home beyond their parents’ observations, which will also enhance parent/child agreement. However, further research exploring daily activities of children with severe SLI is required. The recruitment of a control group allowed for confirmation of any significant differences between mean scores of the control and experimental group. In addition, the cut-off scores proposed by Varni et al. (2003) may vary between countires, since scores 1SD below the mean for this control group were all higher than the published cut-off scores [8]. However, to make firm conclusions, further research on a larger sample of Australian children is required. All the mean scores for both the child self-report and parent proxy-report for children with severe SLI, fell below 1SD of the control group, with only one exception; the emotional functioning scale. Whereas nearly half of the mean scores on both the child self-report and parent proxy-report fell above the cut-off scores published by Varni et al. (2003). Therefore, the recruitment of a control group provided additional clarity on the extent of perceived problems, which suggests that future research with particpants residing outside The United States of America may benefit from the recruitment of a local control group vs utilizing the published cut-off scores when exploring the results of the PedsQL™.

The results of this study are concerning as they imply that a severe SLI has the capacity to signficantly affect a child’s HRQOL negatively overall, and across a variety of domains extending beyond those immediately impacted by the communication difficulties themselves, particularly when compared to age and gender-matched peers. It is becoming accepted that the ultimate goal of healthcare is not only reducing impairments, but also improving an individual’s HRQOL, particularly in chronic health conditions [19]. Since this study provides evidence of the extent that both a child with severe SLI and their parent perceives a language impairment impacts a child’s HRQOL, it is paramount that exploration of HRQOL becomes an integral part of the assessment of these children. Further, this is the first study to identify that a severe SLI significantly impacts negatively on functioning across multiple domains of HRQOL when compared to their peers which may be attributed to the multiple comorbidites experienced by these children [22, 23]. Health professionals, teachers and policy makers need to understand the multifaceted presentation of these children and how their difficulties collaboratively impact on daily functioning. In addition, the outcomes of this study further support the need for a multidisciplinary team to thoroughly assess and identify any developmental challenges endured by these children to ensure holistic intervention to address the diverse needs of these children extending beyond communication.

When compared to other children with developmental disabilities such as Attention-Deficit and Disruptive Behaviour Disorders and Pervasive Developmental Disorders [24] for both the child self-report and parent-proxy report; children with severe SLI scored themselves comparably with the exception of a lower score on the physical functioning scale, and better score on the emotional functioning scale. Parents of children with Asperger’s Syndrome [16] rated their children lower than parents of children with severe SLI. Therefore, it appears that developmental disorders may similarly yet uniquely impact on a child’s HRQOL.

The children included in this study attended a dedicated school, which could be seen as a limitation of this study. However, this study provides evidence that severe SLI significantly impacts negatively on the HRQOL of children even in a protected environment, warranting the need to explore the HRQOL of children with SLI attending mainstream schools. A small sample size, along with a higher porportion of males and younger children was a limitation of this study. However, the percentage of males is representative of the preponderance of males diagnosed with severe SLI [2, 3]. Further, this study provides preliminary evidence of the impact SLI has on the HRQOL of children with severe SLI compared to their peers, while confirming the sutiablity of a HRQOL measure for use with typically developing Australian children.

## Conclusions

Children with severe SLI require a multi-professional assessment that optimally includes evaluating a child’s HRQOL. Australian government policies supporting children with disabilities needs to be updated to reflect the multifaceted presentation of children with severe SLI, particularly because of the impact these associated impairments have on these children’s lives. With the support of the government to provide resources to parents, teachers and health professionals working with children with severe SLI, these children can acquire the support they require to foster optimal development across all domains of life; ultimately facilitating the child with severe SLI to participate in age-appropriate activities and experience happiness in childhood comparable to their peers.

## Abbreviations

HRQOL: Health-related Quality of Life
PedsQL™: Pediatrics Quality of Life Inventory™ 4.0 Generic Core Scales
SLI: Specific language impairment
